# Exploring the effects of *Xinnaoning* capsule in microcirculatory dysfunction model rats through laser speckle contrast imaging and metabolomics

**DOI:** 10.3389/fphar.2025.1647514

**Published:** 2025-08-11

**Authors:** Yuanfang Sun, Shasha Li, Lijing Du, Xiaopeng Ren, Huizi Jin, Shikai Yan, Xue Xiao

**Affiliations:** ^1^ Guangdong Metabolic Diseases Research Center of Integrated Chinese and Western Medicine (Institute of Traditional Chinese Medicine), Guangdong Pharmaceutical University, Guangzhou, China; ^2^ School of Pharmacy, Shanghai Jiao Tong University, Shanghai, China; ^3^ The Second Clinical College of Guangzhou University of Traditional Chinese Medicine, Guangzhou, China

**Keywords:** *Xinnaoning* capsule, microcirculatory dysfunction, laser speckle contrast imaging, metabolomics, pharmacological effects

## Abstract

**Background:**

Microcirculatory dysfunction leads to a number of diseases and worsen prognosis. Blood-activating drugs like *Xinnaoning* capsule may improve circulation, but its pharmacological effects in microcirculatory dysfunction remain unclear.

**Objective:**

The purpose of this study is to determine the pharmacological effect of *Xinnaoning* capsules in treating microcirculatory dysfunction.

**Methods:**

Adrenaline and icy water stimuli were used to establish a rat model of acute microcirculatory dysfunction. Detecting laser speckle contrast imaging, coagulation function, hemorheology, and the Elisa assay were conducted to investigate the effect of *Xinnaoning* capsule on microcirculation in rats. In addition, the untargeted metabolomics was applied to character the therapeutic effect of *Xinnaoning* systematically from the perspective of endogenous terminal metabolites.

**Results:**

Laser speckle contrast imaging showed that model rats suffered low perfusion in ears, feet and tails, and *Xinnaoning* capsule treatment increased microcirculatory blood flow. *Xinnaoning* capsule diminished the reduction of thrombin time, prothrombin time, activated partial thromboplastin time and the elevated fibrinogen level caused by acute microcirculatory dysfunction. *Xinnaoning* capsule could recover the increased blood viscosity as well as the abnormal vasomotor and microcirculation function in model rats. Furthermore, *Xinnaoning* capsule intervention could alter metabolic state in model rats, which was characterized by the abnormality metabolites mainly in pathway of phospholipids and arachidonic acid metabolism.

**Conclusion:**

The macroscopic image and microscopic indicators elucidated that *Xinnaoning* capsule was highly effective against microcirculatory dysfunction. The present study provided a new perspective on the clinical application of *Xinnaoning* capsule, and contributed to explore novel therapeutic drug against microcirculatory dysfunction.

## 1 Introduction

The microcirculation, which encompasses the arterioles, capillaries and venules, serves as the terminal vascular network within the systemic circulation (Guven et al., 2020). It plays a crucial role in facilitating the exchange of substances between the blood and tissue compartments, thereby supplying oxygen and nutrients to meet the metabolic needs of cells throughout the body. This process is regulated by the microcirculation’s ability to modulate vascular permeability and release vasoactive substances (Scalia, 2013). The assessment of microvascular function is a significantly investigation in cardiovascular disease research and risk stratification (Tibiriçá et al., 2018; Crea et al., 2022). Microcirculation is the location where the initial indications of cardiovascular disease, particularly inflammatory reactions, arise, which has a significant impact on the progression of atherosclerosis in conduit vessels (Lockhart et al., 2009). Consequently, microcirculatory dysfunction has emerged as a novel therapeutic focus in the realm of vascular disease prevention.

In pathological conditions such as inflammatory reactions and metabolic disorders, the compromised structure and function of microcirculation hinder the adaptation to the metabolic demands of tissues and organs, thereby impacting material exchange and organ function (Moore et al., 2015). The pathogenesis of metabolic disorders primarily involves hemodynamics, hemorheology, and microangiopathy (Joffre et al., 2020). Based on the pathogenesis of microcirculatory dysfunction, drugs for dilating blood vessels, improving hemorheology and protecting the vascular endothelium have gained extensive usage in clinical settings (Miranda et al., 2016). While pharmaceutical interventions offer symptomatic relief for various aspects of metabolic disorders, addressing the pathophysiological complexity of the disease necessitates a more comprehensive approach beyond targeting individual mechanisms. Traditional Chinese Medicine (TCM) has emerged as a distinctive therapeutic approach for addressing intricate systemic ailments, owing to its multi-component, multi-target, and multi-mechanism attributes, exhibiting considerable promise in the treatment of microcirculatory dysfunction (Sun et al., 2022; Wang et al., 2022).

According to traditional medical theory, microcirculatory dysfunction is classified as a manifestation of blood stasis syndrome, primarily attributed to inadequate blood flow (Yang et al., 2022). Consequently, the main approach in clinical practice for addressing such conditions involves interventions aimed at enhancing blood circulation and alleviating blood stasis (Yang et al., 2023). Research indicates that this treatment strategy effectively widens blood vessels, decreases blood viscosity, and alleviates vasospasm, ultimately improving abnormal hemorheology and yielding significant benefits in the management of coronary heart disease and microcirculatory dysfunction (Han et al., 2017). Several studies have provided evidences that *Ginkgo biloba* L*.* and *Salvia miltiorrhiza* Bunge possess the capacity to improve microcirculation while minimizing adverse effects (Tian et al., 2017; Sun et al., 2020).

*Xinnaoning* capsule (XNN), a commonly used traditional Chinese medicinal formula, is made from five medicinal herbs, including *G. biloba* L. [Ginkgoaceae; Ginkgonis Folium], *Buxus sinica* (Rehder & E.H.Wilson) M.Cheng var. *parvifolia* M.Cheng [Buxaceae; Buxi Folium (Microphyllae)], *S. miltiorrhiza* Bunge [Lamiaceae; Salviae miltiorrhizae radix et rhizoma], *Cinnamomum migao* H.W.Li [Lauraceae; Fructus Cinnamomum migao] and *Allium macrostemon* Bunge [Amaryllidaceae; Allii Macrostemonis Bulbus] (see Supplementary for the preparation method and process). It has functions of promoting circulation of *Qi* and blood, relieving pain and obstructions of collateral vessels, and is widely prescribed for patients suffering from atherosclerotic cardiovascular and cerebrovascular diseases, such as coronary heart disease, angina pectoris, atherosclerosis, cerebral infarction, cerebral insufficiency vertigo, and cerebral stroke (Li et al., 2022; Zhao et al., 2019; Zhu et al., 2021). A multicenter randomized controlled trial has substantiated the safe and effective application of XNN in the treatment of chronic stable angina pectoris complicated with *Qi* stagnation and blood stasis syndrome (Li et al., 2022). It is important to highlight that XNN has demonstrated benefits in improving myocardial microcirculation and cerebral blood circulation, reducing blood lipid levels, and ameliorating cerebral ischemia-reperfusion injury, as evidenced by previous studies. However, the existing literature lacks investigation into the effects of XNN on microcirculatory dysfunction.

This study aims to investigate the pharmacological effect of XNN on microcirculatory dysfunction and gain insight into the underlying effect of XNN on vascular diseases. The pharmacological effect of XNN was determined on an acute microcirculatory dysfunction (AMD) rat model by measuring hematological indicators such as blood perfusion volume and blood viscosity, as well as cytokine levels. Additionally, laser speckle contrast imaging and metabolomics were employed to elucidate the pharmacological mechanisms of XNN on microcirculatory dysfunction, providing insights from both macroscopic imaging characterization and microscopic endogenous metabolites. This integrated methodology is anticipated to offer a thorough and systematic comprehension of the effectiveness of XNN.

## 2 Materials and methods

### 2.1 Chemicals and reagents

*Xinnaoning* capsule (Guizhou Jingcheng Pharmaceutical Co., Ltd., China; Lot: 20201135). Acetylsalicylic acid (ASA) (Dalian Meilun Bio Co., Ltd., China; Lot: D1222A) was prepared into suspension with water. Epinephrine hydrochloride injection (EHI) (Grandpharma China Co., Ltd., China; Lot: 210,505) was provided by Guangdong Provincial Hospital of Chinese Medicine. Activated partial thromboplastin time (APTT) reagent, CaCl_2_ solution, prothrombin time (PT) reagent, thrombin time (TT) reagent, fibrinogen (FIB) reagent and buffer solution were purchased from Chengdu Excellent Medical Scientific Co., Ltd (China). Urethane (Shanghai Aladdin Biochemical Technology Co., Ltd., China), formic acid (HPLC grade, Guangzhou Chemical Reagent Factory, China), methanol (HPLC grade, Merck, Germany), and acetonitrile (HPLC grade, Merck, Germany) were used during the experiment.

### 2.2 HPLC-DAD

The 1.00 g XNN powder was precisely weighed and extracted with 10 mL methanol via ultrasonication (30 min). After cooling, the extract was replenished to the initial weight with methanol, then was centrifuged (12,000 × g, 10 min) and filtered through 0.45 μm membranes prior to analysis.

Chemical profiling of XNN was conducted using an Agilent 1200 high-performance liquid chromatography (HPLC) system coupled with a diode array detector (DAD). The HPLC-DAD analysis was performed on a Phenomenex Luna C_18_ column (4.6 × 250 mm, 5 μm) at 30 °C with acetonitrile (A) and 0.1% (v/v) formic acid-water (B) as mobile phases. The gradient program was as follows, 0–17 min, 3→15% A; 17–28 min, 15→21% A; 28–38 min, 21% A; 38–56 min, 21→39% A; 56–63 min, 39→47% A; 63–69 min, 47→60% A; 69–80 min, 60→80% A; 80–90 min, 80→85% A; 90–95 min, 85% A, with 0.8 mL/min flow rate and 5 μL injection volume. Ultraviolet detection wavelengths were set at 266 nm at 0–90 min.

### 2.3 Animals and treatments

Sixty-six specific pathogen-free male Sprague Dawley (SD) rats (8–10 weeks, 350 ± 20 g) were provided by Guangdong Vital River Laboratory Animal Technology Co., Ltd. (No. 44829700005114). The protocol for animal study was approved by the Ethical Committee of Guangdong Provincial Hospital of Chinese Medicine (No. 2021011). Male SD rats were housed in the experimental animal center of the Guangdong Provincial Hospital of Chinese Medicine under standard environmental conditions (23 °C ± 2 °C, 55% ± 5% humidity and 12 h/12 h light/dark cycle). All rats were allowed free access to water and standard chow.

Rats were randomly divided into six groups, including control (CON, n = 10), model (MOD, n = 12), positive-control (POS, n = 11), XNN low-dose administration (XNN-L, n = 11), XNN medium-dose administration (XNN-M, n = 11) and XNN high-dose administration (XNN-H, n = 11) groups. Rats in the POS group were intragastric administrated with 0.1 g/kg/d ASA solution, and XNN groups were intragastric administrated with XNN solution at varying doses (low, medium and high doses are, respectively, 0.22, 0.43 and 0.86 g/kg/d). Rats in CON and MOD groups were administrated with 10 mL/kg/d water.

### 2.4 Rats model of acute microcirculatory dysfunction

The day after the final gavages, rats in MOD, POS, and XNN groups were injected subcutaneously with 0.8 mg/kg EHI. Two hours later, the animals were forced swimming in icy water for 4 minutes, and rats were re-injected with EHI 2 hours later. Rats assigned to the control group were administered vehicle (0.9% saline) at matching volumes, frequencies, and routes of administration as experimental groups. All rats were fasted overnight before the end of the experiment.

### 2.5 Sample collection

Twelve hours after model establishment, rats were anesthetized by intraperitoneal injection of urethane (1.5 g/kg). Subsequently, blood samples were obtained from the abdominal aorta using blood collection tubes containing sodium citrate, heparin, or no additives. Blood samples collected in sodium citrate-anticoagulated tubes were centrifuged (1,500 × g, 10 min) to obtain plasma for coagulation assays, while heparinized tubes yielded plasma for hemorheological analysis, and clotted serum from activator-containing tubes was allocated to cytokine quantification and untargeted metabolomics, with all aliquots stored at −80 °C until analysis.

### 2.6 Pharmacological effects evaluation

#### 2.6.1 Laser speckle contrast imaging

PeriCam PSI System was used to conduct laser speckle contrast imaging (LSCI). 785 nm laser was used for blood perfusion measurements, and 2,448 × 2048 pixels CCD camera capable of 120 frames per second was used to monitor the speckle pattern in the illuminated area. To facilitate the alignment of the imager, visible red laser (650 nm) was applied to demarcate the maximum measurement region at appropriate measurement distance. Rats were positioned on a level surface, ensuring that the distance between the laser beam and the surface of the foot (or ear or tail) was precisely 100 mm. A dedicated camera was utilized for documentation purposes, and an image frame measuring 40 mm in width and 30 mm in height was chosen. The sampling frequency was set as 16 Hz, and the average of five consecutive images was computed to yield an effective frame rate of one image per second. Pseudo-color images were generated to represent perfusion levels, with blue indicating low perfusion and red indicating high perfusion. The PimSoft software (version 1.5) was employed to analyze real-time graphs and measure regions of interest (ROIs) in the feet, ears, and tails of rats. The semiquantitative perfusion unit and area were averaged during the sampling period. The mean perfusion was calculated using the PimSoft software.

#### 2.6.2 Coagulation function detection

Blood samples were centrifuged at 3,500 rpm for 10 min in sodium citrate vacuum tubes, and subsequently placed in the sample rack of an EC680 automatic coagulation analyzer (Chengdu Excellent Medical Scientific Co., Chengdu, China).

Firstly, the auto-coagulation analyzer aspirated 50 μL of plasma into the sample cup and maintained it at a temperature of 37 °C for duration of 1 min. Subsequently, 50 μL of APTT reagent was added and thoroughly mixed with the plasma. The resulting mixture was allowed to incubate for an additional 1 min, after which 50 μL of 0.025 mol/L CaCl_2_ solution was introduced. The activated partial thromboplastin time was then recorded. Secondly, another 50 μL of plasma was aspirated into the sample cup and incubated at a temperature of 37 °C for 3 min. This plasma was then mixed with 100 μL of PT reagent and the resulting prothrombin time was recorded. Thirdly, 100 μL of plasma was aspirated and preheated to 37 °C. Subsequently, it was mixed with 100 μL of TT reagent in order to measure the coagulation time. Finally, 200 μL of plasma was diluted with a buffer solution at a ratio of 1:10 and subjected to incubation at 37 °C for 3 min. Subsequently, 100 μL of thrombin reagent was introduced to assess the concentration of fibrinogen (FIB).

#### 2.6.3 Hemorheology detection

The blood collection tubes containing heparin were positioned within the sample receptacle of a MEN-C100A blood rheometer (Jinan Meiyilin Electronic Instrument Co., Ltd., Jinan, China), which facilitated the measurement of blood viscosity at high, medium, and low shear values. The plasma viscosity was subsequently determined by subjecting the samples to centrifugation at 3,500 rpm for a period of 10 min.

#### 2.6.4 ELISA assays

ELISA kits for the detection of vascular endothelial growth factor (VEGF), endothelin-1 (ET-1), nitric oxide synthase (NOS), intercellular adhesion molecule-1 (ICAM-1), vascular-endothelial cell cadherin (VECD), cyclooxygenase-2 (COX-2), thromboxane B2 (TXB2) and prostacyclin (PGI2) were procured from Shanghai Enzyme-linked Biotechnology Co., Ltd. The detection procedure was carried out in accordance with the instructions provided by the kits.

### 2.7 Metabolomics

#### 2.7.1 Sample preparation

Frozen serum samples were thawed at a temperature of 4 °C, followed by transferring 200 μL of serum to a sterile tube and mixing it with a tripled quantity of methanol. The resulting mixture was vortex for 1 min and static settled at 4 °C for 30 min, following centrifugation at 14,000 rpm for 10 min. The supernatant was carefully transferred to a sterile tube and filtered with 0.22 μm microporous membrane for metabolomics analysis. QC were prepared by combining an equal volume of serum from each sample and analyzed at every eight injection intervals during the operation.

#### 2.7.2 Serum metabolomics analysis

Chromatography analysis was conducted using a Waters ACQUITY UPLC system (Waters, USA). Serum samples were performed on an ACQUITY UPLC BEH C_18_ column (2.1 mm × 100 mm, 1.7 μm) maintained at 35 °C. The mobile phase consisted of 0.1% formic acid dissolved in water (mobile phase A) and methanol (mobile phase B). A gradient elution was used in the process: 0–5 min: 5%→30% B; 5–35 min: 30%→95% B; 35–46 min: 95%B (isocratic). An 8-min equilibration period was implemented between samples to restore the initial conditions. The flow rate was set at 0.3 mL/min, and the injection volume was 2 μL.

Mass spectrometry analysis was conducted using a quadrupole time-of-flight mass spectrometer (AB SCIEX, USA) with an electrospray ionization ion source. Both positive and negative ionization modes were investigated in this study and abundant chromatographic peaks can be detected with acceptable signal-to-noise ratio in positive ionization mode. Therefore, positive (ESI+) ion mode is finally adopted in this study.

The flow rate of ion source gas one and ion source gas two were set at 50 psi, and the curtain gas was maintained at 35 psi. The capillary voltage for the ion source was adjusted to 5500 V at a temperature of 500 °C, and the declustering potential voltage was 80V. TOF-MS survey scan was conducted in the range of 100–2000 Da, followed by 6 M/MS scans in the range of 50–2000 Da. The accumulation times for the TOF-MS and MS/MS scans were 0.25 s and 0.1 s, respectively. The collision energy was 10 V for TOF-MS and 40 ± 20 V for MS/MS. The ion release delay for product ion was 67, and the ion cluster width was 25. Dynamic background subtraction was utilized.

#### 2.7.3 Data processing

The raw LC-MS data were processed using Progenesis QI (Waters, USA) for deconvolution, peak picking, alignment, and area normalization. Significant features were identified by aligning them with features in the reference sample, employing filters with a retention time (RT) window of less than 0.2 min and a mass tolerance of less than 5 ppm. The features were annotated by matching accurate mass and tandem MS data with the human metabolome database (HMDB). A mass tolerance of 5 ppm was applied for both precursor and fragment ions. Metabolites with a fragmentation score below 20 were excluded from annotation. All 3,404 annotated metabolite characteristics were presented in Supplementary Table S1, along with the Progenesis QI score, fragmentation score, and isotope similarity derived from precise mass and fragmentation data.

The processed data was imported into the SIMCA-P + (version 13.0) software package (Umetrics, Umeå, Sweden) for conducting principal component analysis (PCA) and partial least square-discriminant analysis (PLS-DA). PCA was utilized to assess the overall disparity in metabolic profiles between groups, while PLS-DA was subsequently employed to optimize the differentiation in metabolic profiling. The accuracy of the model was evaluated using the value of Q^2^Y and R^2^Y, and a standard 7-round cross validation was performed to validate the model and prevent over fitting. The Variable Important in Projection (VIP) value was calculated and obtained through the use of PLS-DA. Subsequently, the significance of metabolites with a VIP value greater than one was determined using univariate statistical analysis (as shown in Supplementary Material, Supplementary Table S2).

#### 2.7.4 Pathway analysis

The differential expression of metabolites was subjected to pathway analysis using MetaboAnalyst 5.0 (https://www.metaboanalyst.ca). Metabolites were mapped onto the *Homo sapiens* Kyoto Encyclopaedia of Genes and Genomes (KEGG) metabolic network, and the significance of a pathway was assessed through the hypergeometric test, while the impact of the pathway was determined by analyzing the relative between centrality in the topology analysis.

### 2.8 Statistical analysis

Statistical analyses were performed using SPSS software (Version 18.0, USA). Assumptions of normality and homogeneity of variance were first checked. The data were presented as the mean ± standard deviation (SD).

The one-way ANOVA were conducted to analyze the differences among groups for continuous measures. Differences with *p* values less than 0.05 were considered statistical different, and *p* values less than 0.01 was considered significant difference.

## 3 Results

### 3.1 Chemical profiling of XNN

**FIGURE 1:**
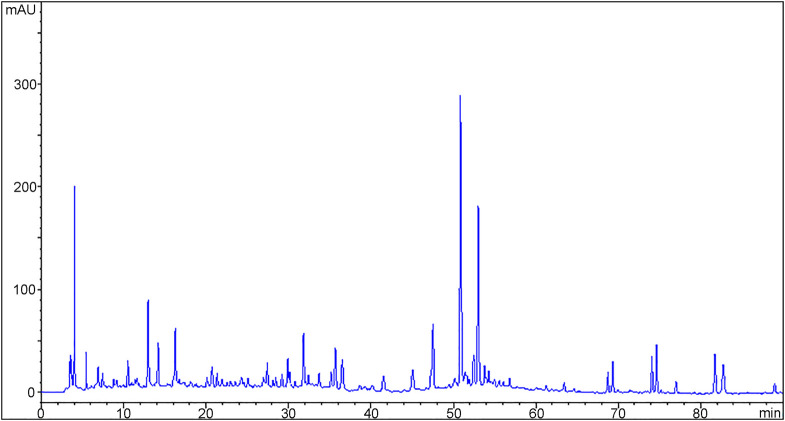
A typical HPLC-DAD fingerprint of *Xinnaoning* capsule acquired on an Agilent 1200 HPLC system with Phenomenex Luna C_18_ column (4.6 × 250 mm, 5 μm) using gradient elution (mobile phase A: acetonitrile, B:0.1% formic acid-water) at 0.8 mL/min flow rate, 30 °C, 5 μL injection volume, and 266 nm detection wavelength.

The pharmacological effect of XNN is attributed to its chemical composition. As a TCM compound preparation made from five medicinal herbs, XNN contains a large number of chemical components, which are mainly derived from the secondary metabolites of herbal materials. Figure 1 shows a typical chemical fingerprint of XNN detected at 266 nm reference wavelength that reflects its overall chemical composition.

The numerous chromatographic peaks in Figure 1 represent the major chemical components in XNN. To further characterize the structural information of the chemical components in XNN, we conducted LC/MS analysis (see Supplementary Material for UPLC/MS analysis method). We identified 35 major chemicals in XNN, including tanshinone VI, salvianolic acid E, rutin, 6-shogaol, linolenic acid, etc., most of which are flavonoids, phenolic acids, and fatty acids (as shown in Supplementary Material, Supplementary Table S3).

### 3.2 XNN increased local blood flow in the AMD rats

**FIGURE 2:**
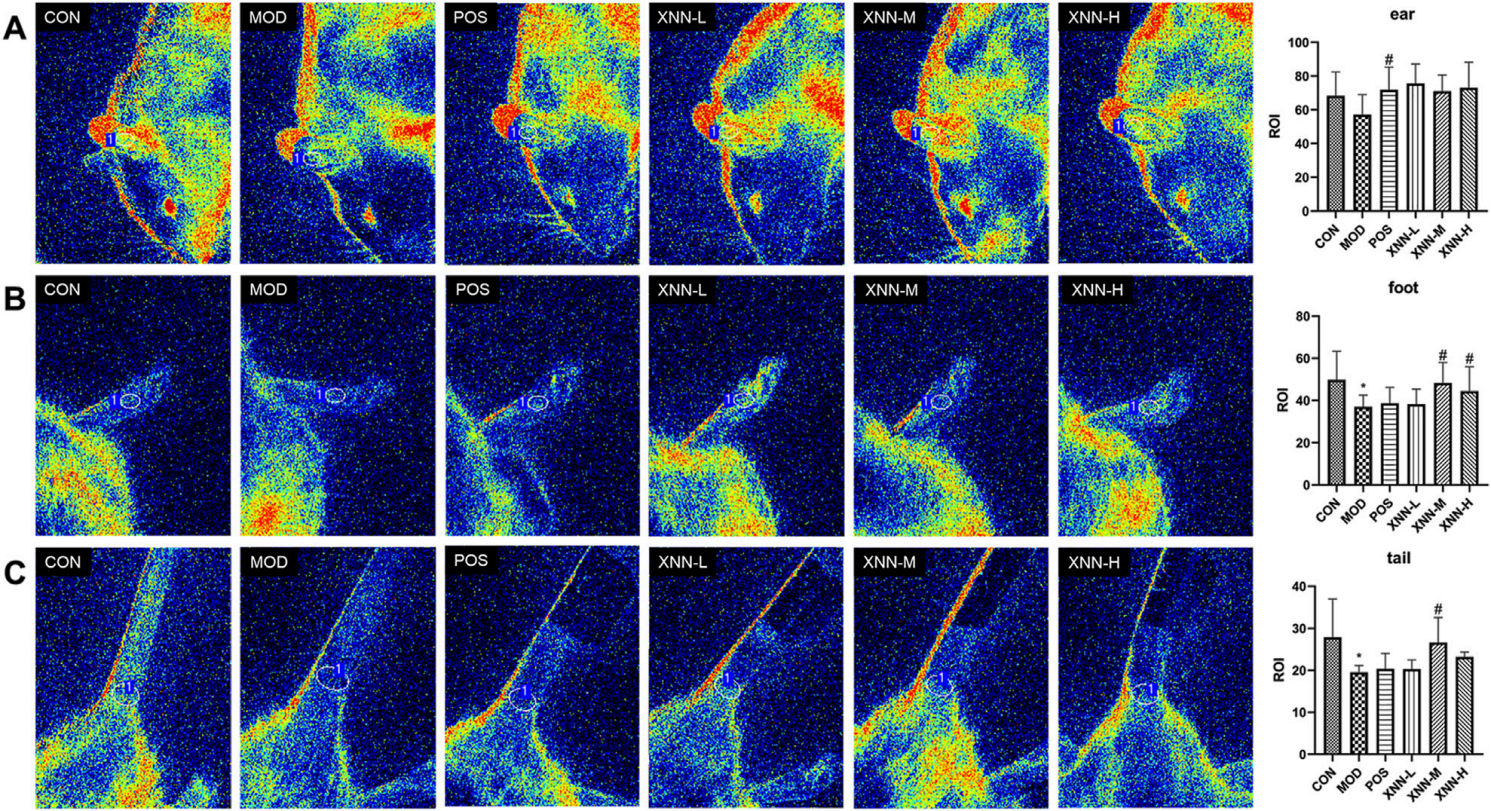
Laser speckle contrast imaging image of rats’ ears **(A)**, feet **(B)** and tails **(C)**. CON, control group (n = 5); MOD, model of acute microcirculation dysfunction group (n = 6); POS, positive control group (n = 6); XNN-L, *Xinnaoning* capsule low-dose administration group (n = 6); XNN-M, *Xinnaoning* medium-dose administration group (n = 6); XNN-H, *Xinnaoning* high-dose administration group (n = 6). Statistical significance indicates as asterisk (*) when comparing CON group with MOD group, and as hashtag (#) when POS, XNN-L/M/H group with MOD group. *: *p* < 0.05; #: *p* < 0.05.

Microcirculatory assessment was performed on a subset of animals at 12 h post-modeling using laser speckle contrast imaging (PeriCam PSI System, Perimed AB). Specifically, rats from the CON group (n = 5), MOD group (n = 6), POS group (n = 6), XNN-L group (n = 6), XNN-M group (n = 6), and XNN-H group (n = 6) were imaged under standardized conditions (ambient temperature 25 °C, anesthetic depth confirmed by pedal reflex test). This pre-terminal imaging was completed prior to blood collection to avoid hemodynamic interference. A region of interest was chosen to ensure the stability of the analyzed area. The speckle contrast values for each frame were spatially averaged, and the statistical processing results of microvascular blood flow changes in the ears, feet, and tails of different groups were presented in histogram format. As depicted in Figure 2, the LSCI analyses demonstrated a notable decrease in microvascular blood flow in the ears, feet, and tails of rats afflicted with AMD in comparison to healthy rats, with statistically differences observed in the feet and tails. Following the administration of ASA and XNN, the local blood flow exhibited an upward trajectory, with statistically significant disparities observed in the ears of POS group, in the feet and tails of the middle-dose XNN group, and in the feet of the high-dose XNN group when compared to the model group. Results indicated that XNN could effectively increase the local blood flow in rats and showed stronger effects on increasing local blood flow in the foot and tail of modeled rats than acetylsalicylic acid.

### 3.3 XNN restored coagulation function in the AMD rats

**FIGURE 3:**
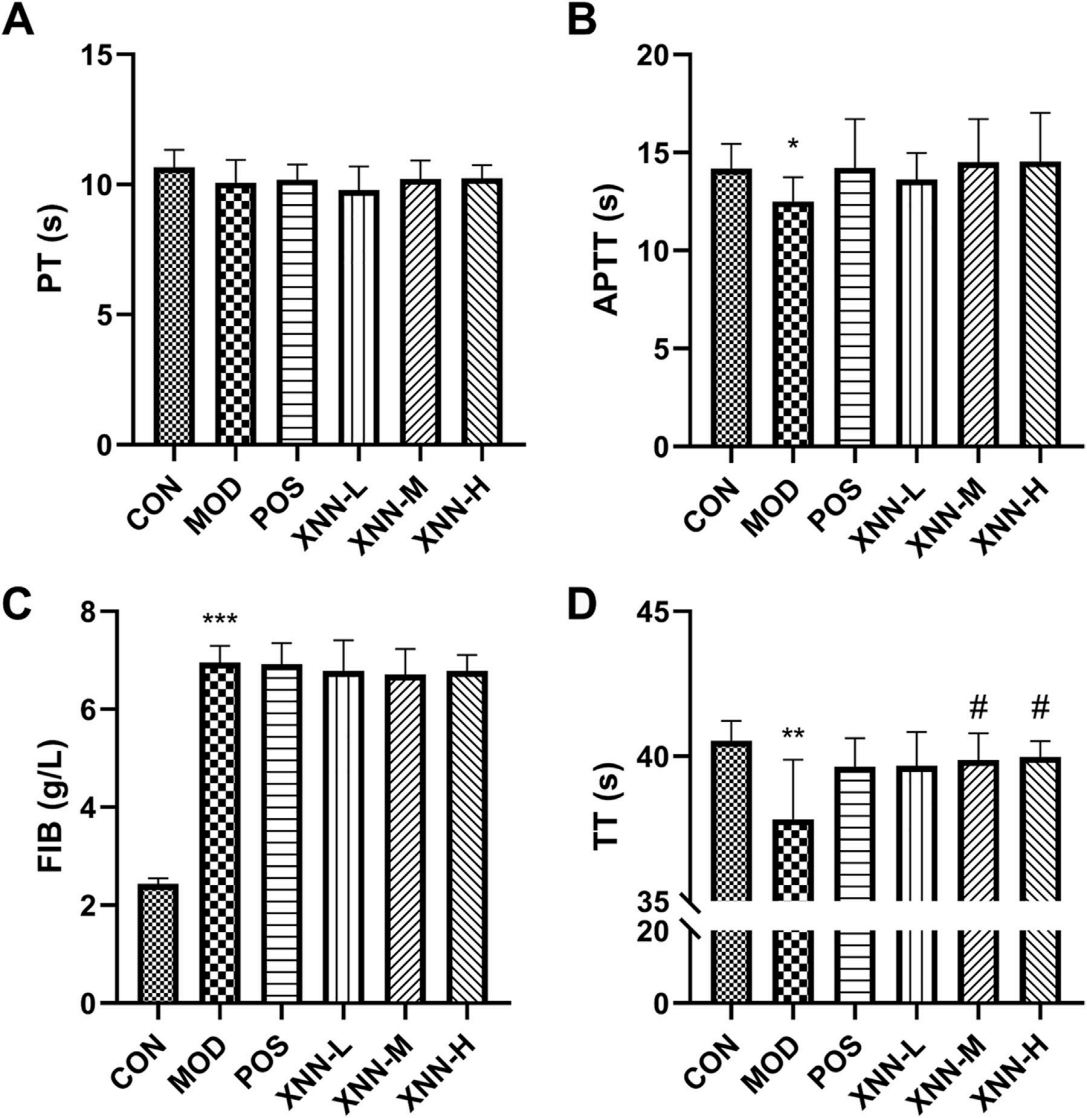
Results of coagulation function test. **(A)**, prothrombin time (PT); **(B)**, activated partial thromboplastin time (APTT); **(C)**; fibrinogen (FIB); **(D)**, thrombin time (TT). CON, control group (n = 5); MOD, model of acute microcirculation dysfunction group (n = 8); POS, positive control group (n = 7); XNN-L, *Xinnaoning* capsule low-dose administration group (n = 7); XNN-M, *Xinnaoning* medium-dose administration group (n = 7); XNN-H, *Xinnaoning* high-dose administration group (n = 7). Statistical significance indicates as asterisk (*) when comparing CON group with MOD group, and as hashtag (#) when POS, XNN-L/M/H group with MOD group. *: *p* < 0.05, **: *p* < 0.01, ***: *p* < 0.001; #: *p* < 0.05.

The impact of XNN on blood coagulation function was assessed by measuring PT, APTT, FIB, and TT levels in the plasma of rats. Figure 3 illustrates a decrease in APTT (*p* < 0.05) and TT (*p* < 0.01), and a significant elevation of FIB level (*p* < 0.001) in AMD rats compared to healthy rats. After administration of XNN and ASA, the APTT, TT and FIB showed a callback trend after intervention without statistical difference, except for the TT in the middle/high dose of XNN group prolonged statistically (*p* < 0.05). This study revealed that pharmacological effects of XNN in improving coagulation function mainly focuses on inhibiting the transformation of fibrinogen into fibrin. In conjunction with the significance of four coagulation indexes, these findings suggest that XNN can effectively modulate the endogenous coagulation function in AMD rats, and XNN plays an equivalent effect to ASA.

### 3.4 XNN improved hemorheology parameters in the AMD rats

**FIGURE 4:**
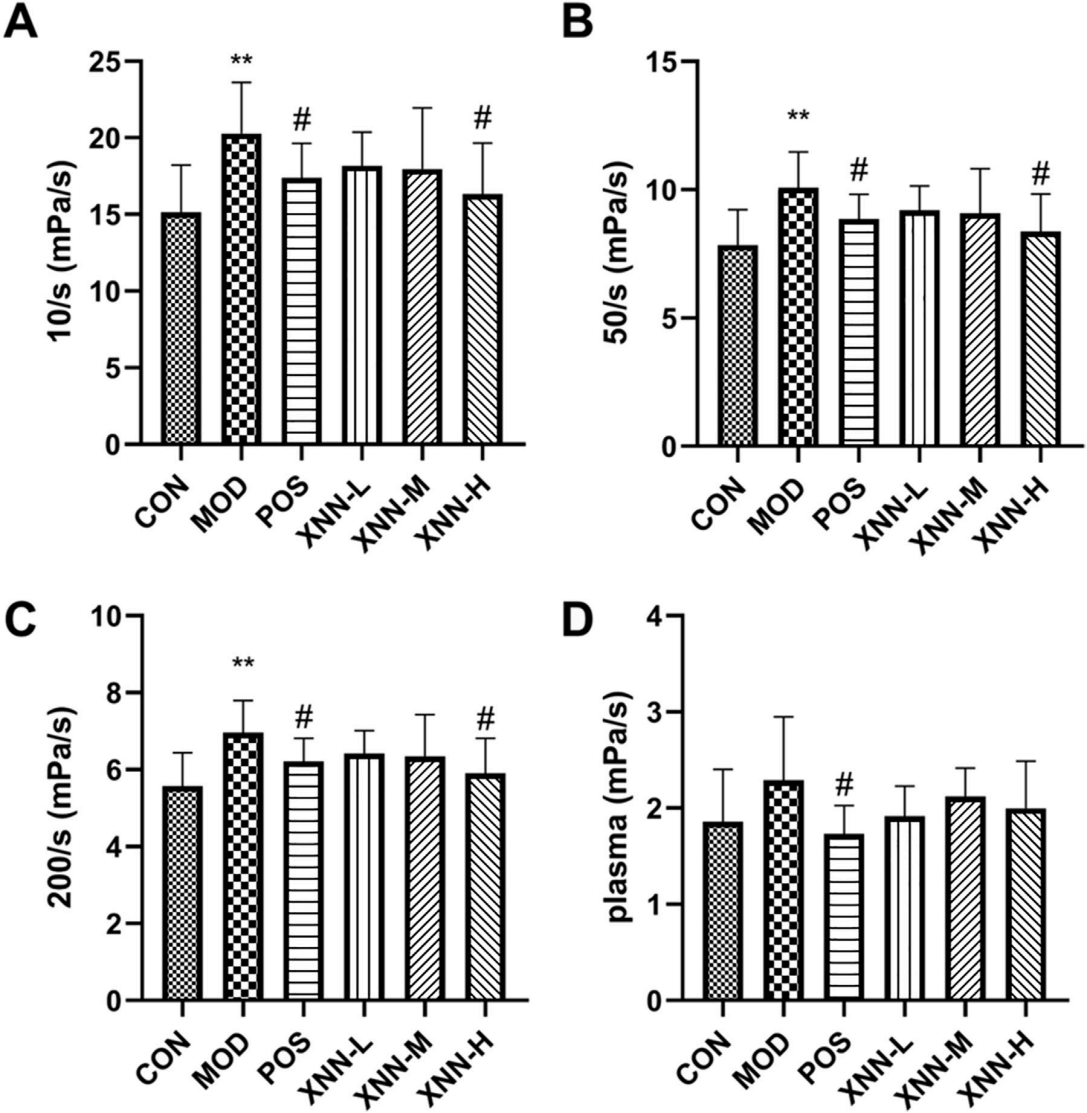
Results of hemorheology parameters detection. **(A)**, whole blood viscosity (WBV) at low shear rate; **(B)**, WBV at medium shear; **(C)**, WBV at high shear; **(D)**, plasma viscosity. CON, control group (n = 7); MOD, model of acute microcirculation dysfunction group (n = 10); POS, positive control group (n = 8); XNN-L, *Xinnaoning* capsule low-dose administration group (n = 7); XNN-M, XNN medium-dose administration group (n = 7); XNN-H, XNN high-dose administration group (n = 7). Statistical significance indicates as asterisk (*) when comparing CON group with MOD group, and as hashtag (#) when POS, XNN-L/M/H group with MOD group. **: *p* < 0.01, #: *p* < 0.05.

Hemorheological detection was conducted on rats, evaluating parameters such as whole blood viscosity (WBV) at low shear rate, WBV at medium shear rate, WBV at high shear rate, and plasma viscosity (PV). According to the findings presented in Figure 4, WBVs of AMD rats exhibited a significant increase after modeling, which differed statistically from the values observed in the normal rats (*p* < 0.01). Following the administration of ASA and XNN, WBVs displayed a trend of improvement, and statistical difference was observed in high-dose XNN group and ASA group. PV of rats increased after modeling and decreased after XNN intervention without statistically significant. These results indicate that XNN effectively alleviates the condition of high blood viscosity in AMD rats, and its function mainly focuses on blood cells.

### 3.5 XNN restored microcirculatory function of the AMD rats

ELISA assays were conducted using plasma from rats in the CON group (n = 7), MOD group (n = 12), POS group (n = 11), XNN-L group (n = 9), XNN-M group (n = 10) and XNN-H group (n = 10) to evaluate the microcirculatory function.

**FIGURE 5:**
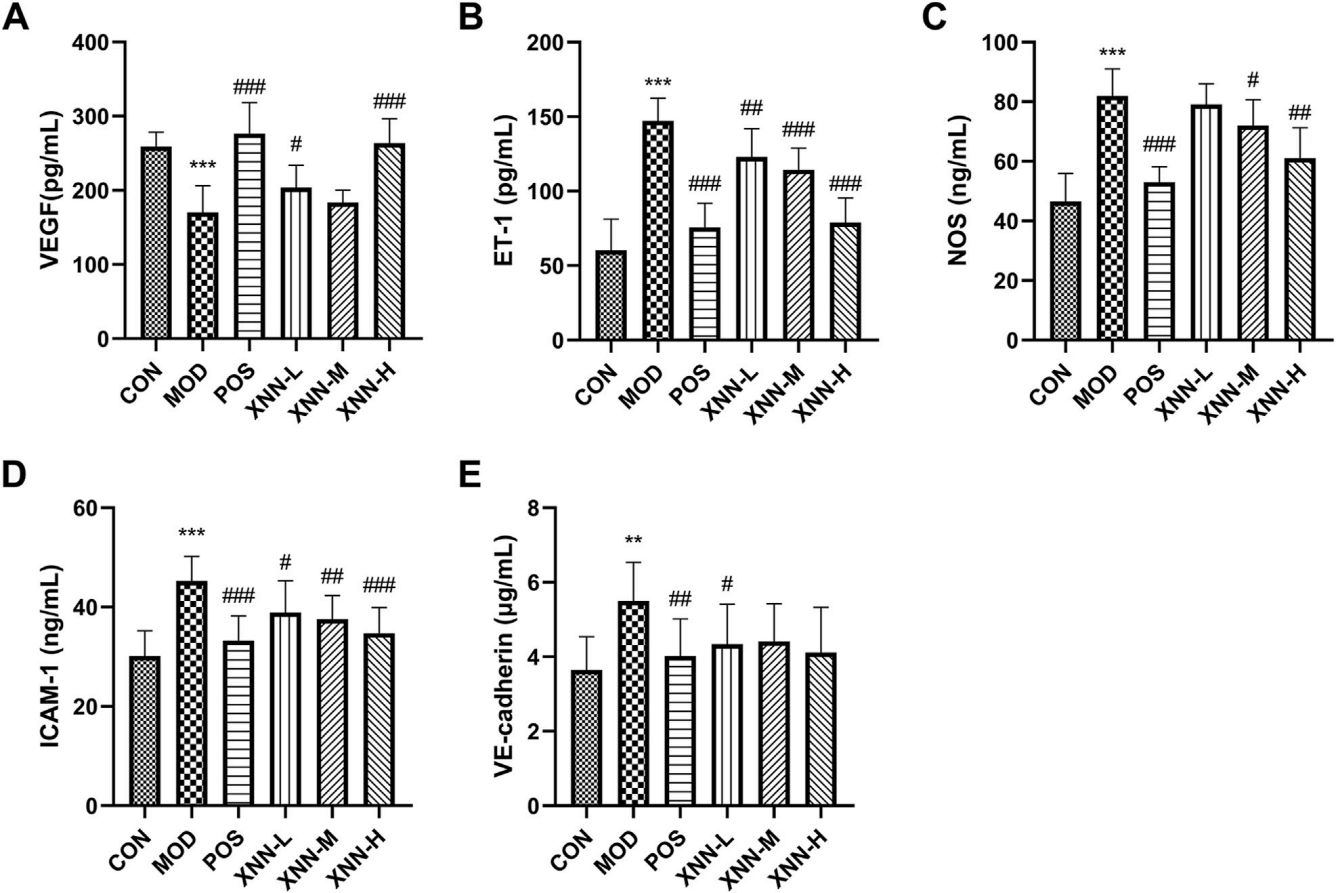
Results of biochemical indexes assay. **(A)**, vascular endothelial growth factor (VEGF); **(B)**,endothelin-1 (ET-1); **(C)**, nitric oxide synthase (NOS); **(D)**, intercellular adhesion molecule-1 (ICAM-1); **(E)**, vascular endothelial-cadherin (VECD). CON, control group (n = 7); MOD, model of acute microcirculation dysfunction group (n = 12); POS, positive control group (n = 11); XNN-L, *Xinnaoning* capsule low-dose administration group (n = 9); XNN-M, *Xinnaoning* medium-dose administration group (n = 10); XNN-H, *Xinnaoning* high-dose administration group (n = 10). Statistical significance indicates as asterisk (*) when comparing CON group with MOD group, and as hashtag (#) when POS, XNN-L/M/H group with MOD group. **: *p* < 0.01, ***: *p* < 0.001; #: *p* < 0.05; ##: *p* < 0.01; ###: *p* < 0.001.

According to the findings presented in Figure 5, the level of VEGF in modeled rats exhibited a significant decrease compared to the control group (*p* < 0.001). After administration of ASA or XNN, the serum VEGF level increased, with the high dose groups of ASA and XNN showing a recovery to normal levels. These results suggest a positive impact of XNN on enhancing the function of vascular endothelial cells, with the high dose of XNN demonstrating an equivalent effect to ASA.

NO derived from NOS is an endogenous vascular relaxing factor, which can regulate vascular tone and endothelial continuity together with endothelin-1, effectively relax blood vessels and increase blood flow. In this study, ET-1 and NOS were detected to evaluate vasoconstriction and vasodilatation. As a result, the levels of ET-1, and NOS were found to be significantly increased in the models (*p* < 0.001), and this increase was significantly ameliorated by ASA and XNN. The impact of XNN on improving vasomotor function was found to be positively correlated with the drug dose, and the effect of high dose XNN was found to be similar to ASA. These changes observed in serum suggest that XNN can effectively improve the vasomotor function of the AMD model.

ICAM-1 and VECD were utilized as indicators to assess the vascular permeability of rats. Notably, the serum levels of ICAM-1 and VECD in model rats exhibited a significant increase in comparison to those of normal rats, which subsequently decreased following drug administration (*p* < 0.01). The effect of XNN treatment displayed some positive correlation with the dosage, with the high dosage exhibiting a similar impact to ASA. Particularly, XNN exhibited the most pronounced influence on the VECD level. Various doses of XNN effectively reduced the serum content of VECD in AMD model rats, with no statistically significant difference observed when compared to the control group. The data presented in this study demonstrates pharmacological effects of XNN in improving vascular permeability in rats with AMD.

### 3.6 XNN influenced metabolic state in the AMD rats

**FIGURE 6:**
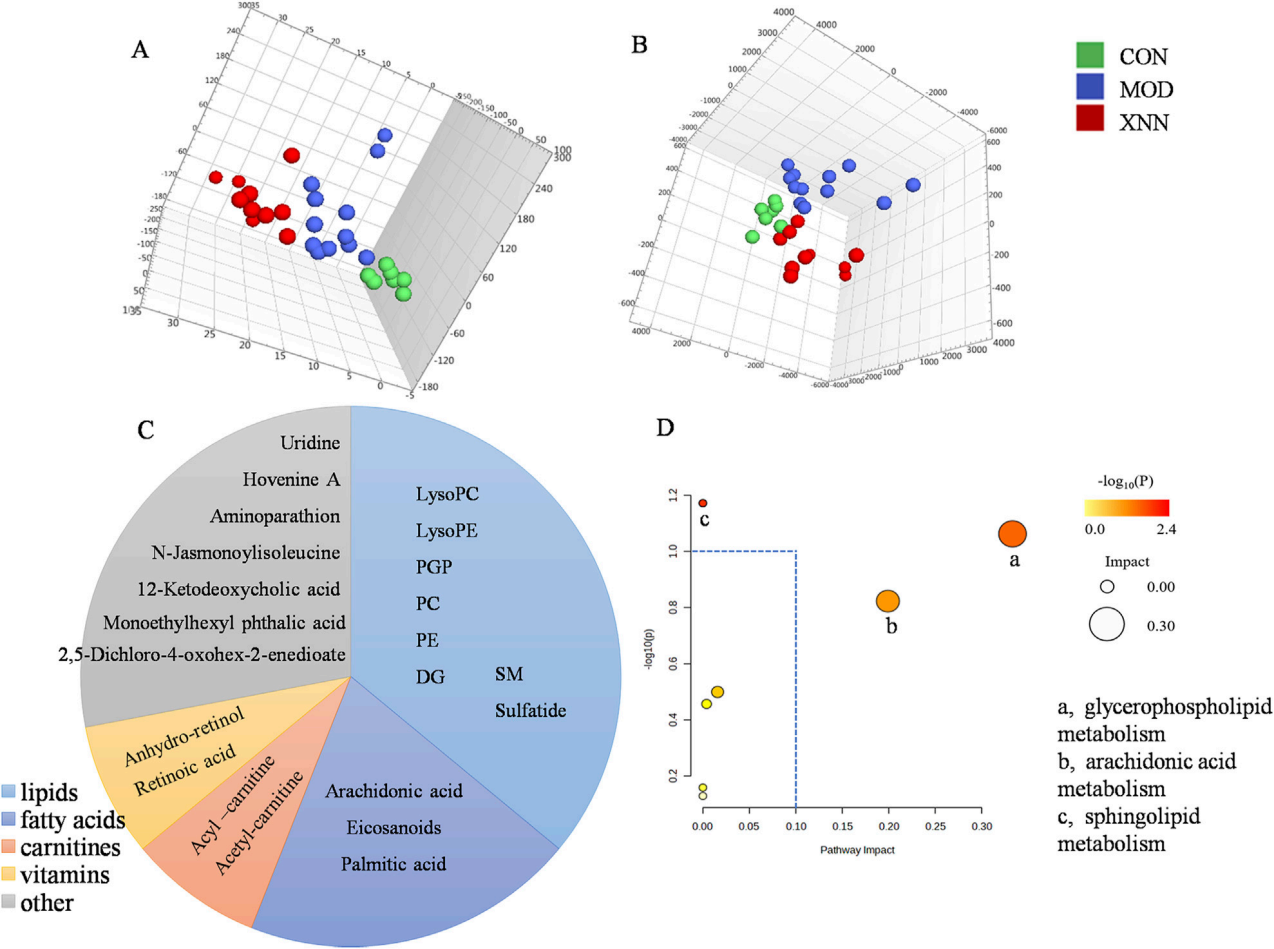
Results of metabolomics analysis of rat’s plasma from control group (n = 7), model of acute microcirculation dysfunction group (n = 12), *Xinnaoning* high-dose administration group (n = 10). **(A)**, 3D scores plot of principal component analysis; **(B)**, 3D score plot of partial least squares discriminant analysis; **(C)**, Classification of potential biomarkers; **(D)**, Pathway analysis: a represents glycerophospholipid metabolism, b represents arachidonic acid metabolism, c represents sphingolipid metabolism.

In order to investigate the underlying mechanism of XNN’s effect on microcirculatory dysfunction, an untargeted metabolomics analysis of rat serum from CON group (n = 7), MOD group (n = 11) and XNN-H group (n = 10) was conducted. Metabolic spectrums of serum samples were obtained using UPLC-Q/TOF MS. A regression model of PCA was constructed to predict variations among CON group, MOD group and XNN-H group. PCA model score plots (Figure 6A) clearly indicate a distinct separation between the CON group and MOD group, suggesting that the metabolomic profile in the serum of rats with AMD differs significantly from that of healthy rats. The findings demonstrate the advantage of metabolomics in characterizing the physiological state of rats with AMD, and highlight the potential of XNN in rectifying the metabolic irregularities in the animal model.

### 3.7 XNN changed metabolites expression in the AMD rats

To elucidate the differential metabolites among the CON group, MOD group, and XNN-H group, we constructed PLS-DA models to visually represent the metabolic disparities (Figure 6B). A distinct inclination towards differentiation was observed between AMD and XNN-H.

The VIP score of the PLS-DA model demonstrated the significance and explanatory capability of metabolite expression patterns in classifying and distinguishing samples from each group. Based on the criteria of VIP>1.0 and *p* < 0.05, a total of 26 metabolites exhibited differential expression in serum among the CON group, MOD group, and XNN-H group (Figure 6C). As shown, in addition to high-abundance lipids (including glycerophospholipid, glyceride, sphingolipid), fatty acids (including arachidonic acid, eicosanoids and palmitic acids), carnitines, vitamins were also identified as differential metabolites.

Subsequently, a pathway analysis was performed on the 25 differentially expressed metabolites using the MetPA (metabolomics pathway analysis) approach in MetaboAnalyst 5.0. The metabolite data was imported into Pathway Analysis to assess the significance of metabolic pathways, resulting in the enrichment of seven metabolic pathways. Notably, arachidonic acid metabolism, glycerophospholipid metabolism and sphingolipid metabolism were prominently highlighted, as depicted in Figure 6D.

## 4 Discussion

The microcirculation, which serves as the terminal vascular network of the systemic circulation, represents the ultimate destination of the cardiovascular system. Arguably, the microcirculation holds utmost significance within the cardiovascular system as it bears the responsibility of delivering oxygen to fulfill energy demands, as well as facilitating the transportation of blood-borne hormones and nutrients to tissue cells (Se et al., 2011). Notably, microcirculatory dysfunction not only contributes to the development of various chronic diseases and metabolic disorders, but also holds relevance in the realm of acute and critical illnesses (Mejía-Rentería et al., 2019). Sudden death frequently arises in cases of capillary spasms or atresia, as well as during the stage of pronounced capillary stasis, blood flow deceleration, and micro emboli formation (El-Maraghi and Genton, 1980). It is evident that managing microcirculatory dysfunction could offer novel insights to clinicians in addressing clinical challenges.

XNN represents a natural pharmaceutical formulation, and is frequently prescribed in clinical settings for the treatment of atherosclerotic cardiovascular diseases and has demonstrated effect in improving microcirculation. Contemporary pharmacological investigations have demonstrated that *G. biloba* L. functions as an inhibitor of the platelet-activating factor and a vasodilator (Tian et al., 2017) and *A. macrostemon* Bunge exhibits anti-platelet aggregation, anti-atherosclerotic, cardiomyocyte, and vascular endothelial cell protective effects (Wu et al., 2023). An increasing body of clinical research has reported on their utilization in cardiovascular disease and peripheral vascular disease. *Salvia miltiorrhiza* Bunge*,* a renowned botanical drug, possesses extensive cardiovascular and cerebrovascular protective properties and has been utilized in Asian countries for numerous centuries. The extraction of *S. miltiorrhiza* Bunge*,* has the potential to restore endothelium-dependent vasorelaxation and prevent vascular diseases, specifically atherosclerosis and cardiac diseases (Li et al., 2018; Han et al., 2008). *Buxus sinica* (Rehder & E.H.Wilson) M.Cheng and *Camphora migao* H.W.Li are proven to be effective in the folk medicine for the treatment of cardiovascular diseases, as well as alleviating associated symptoms such as chest pain, chest tightness, and asthma. The present pharmacodynamic study elucidated that XNN administration resulted in increased blood flow in local microvessels of AMD rats, along with prolonged coagulation time, reduced blood viscosity and vascular wall permeability, and enhanced vasomotor function. These findings provide objective data characterizing the therapeutic potential of XNN in addressing microcirculation disorders.

Assessing blood flow within the microvasculature serves as a crucial physiological parameter for evaluating the functionality of the microcirculatory system. In this study, laser speckle contrast imaging was employed to rapidly and non-destructively monitor the blood flow in the ear, foot, and tail of rats (Qureshi et al., 2024). It was observed that the blood flow in all anatomical regions of the rats exhibited a significant reduction following the modeling procedure. Furthermore, the administration of XNN effectively reversed the adverse perfusion of micro-vessel. The findings of this study suggest that the observed elevation in blood viscosity and reduction in local microvascular blood flow in rats with AMD can be primarily attributed to heightened red blood cell aggregation, impaired deformability, and increased fibrinogen concentration in their blood. These factors contribute to accelerated coagulation and heightened resistance to blood flow. The administration of XNN was found to prolong the coagulation time, decrease blood viscosity, effectively restore micro-vessel perfusion in AMD rats, and ultimately ameliorate microcirculation disorders.

Disturbed flow exacerbates endothelial apoptosis (Bai et al., 2010), a critical factor in the preservation of blood fluidity and microcirculation (Neubauer and Zieger, 2022; Bloom et al., 2023). Reductions in localized blood flows contribute to the development of an ischemic/hypoxic milieu, resulting in endothelial cell injury within micro-vessels. The aberrant ndothelial function observed in AMD rats is characterized by diminished VEGF and enhanced level of ICAM-1 and VECD in this experiment. VEGF, a pivotal cytokine for endothelial cell functionality, is an endogenously synthesized vascular cytokine that influences microvascular permeability, angiogenesis, and vasodilation (Apte et al., 2019). ICAM-1 has been found to be involved in the adhesion of neutrophils and monocytes to endothelial cells, which contributes to the infiltration of neutrophils into the myocardium and the occurrence of microvascular coronary slow flow (Benson et al., 2007). Elevated levels of ICAM-1 have been observed in patients with atherosclerosis, heart failure, coronary artery disease, and transplant vasculopathy, as it has been measured in various body fluids (Lawson et al., 2009). VECD, a member of the classical cadherin super family, is a crucial component of cell-to-cell adhesion junctions in endothelial cells and plays a significant role in the regulation of vascular permeability (Rho et al., 2017). The reduction of VEGF and increase of ICAM-1 and VECD in the AMD rats suggests the presence of endothelial dysfunction in the model rats. Following XNN intervention, the concentration of these factors recovered, particularly in the high-dose group, reaching levels comparable to those observed in the normal control group. This finding indicates that XNN has the ability to enhance the endothelial function of AMD rats effectively.

Beyond metabolic dilation, endothelial cell usually releases diverse of biological molecules including NO, other reactive oxygen species, and arachidonic acid metabolites to modulate vascular tone (Gutterman et al., 2016). In metabolomics research, arachidonic acid metabolism, glycerophospholipid metabolism and sphingolipid metabolism, which were closely related to the structure or function of vascular endothelium, have been enriched. The literature demonstrates that arachidonic acid metabolism is essential in the regulation of vascular tone (Berra-Romani et al., 2019). Glycerophospholipids and sphingolipids are crucial components of organelle and cell membranes, enhancing fat absorption and utilization, preventing cholesterol deposition in blood vessels, reducing blood viscosity, promoting blood circulation, and contributing to the prevention of cardiovascular diseases (Jozefczuk et al., 2020; Wasserman et al., 2020). Ceramide, a pivotal metabolite in sphingolipid metabolism, serves as a signaling mediator that regulates vasomotor function by activating endothelial nitric oxide synthase (NOS) and facilitating nitric oxide (NO) generation (Cogolludo et al., 2019). Furthermore, ceramide may potentially play a role in the pathogenesis of endothelial dysfunction by modulating nitric oxide synthesis or promoting its degradation through reactive oxygen species generation (Li and Zhang, 2013). The positive impact of XNN on vasomotor function, blood viscosity, and endothelial dysfunction in AMD rats highlights the regulatory effects of XNN on endogenous metabolites.

Metabolomics analysis indicates that the effect of XNN in AMD may be associated with alterations in levels of COX-2, TXB2, and PGI-2 in the bloodstream, in addition to conventional markers. COX-2 is found in endothelial cells to facilitate arachidonic acid to produce prostaglandin and thromboxane (TXs). Serum TXB2 has been identified as a risk factor for ischemic stroke and ischemic heart disease for its ability to induce vasoconstriction, platelet aggregation, leukocyte adhesion, and promotion of oxidative stress (Szczuko et al., 2021). PGI2 is vasoactive substances synthesized by the normal endothelium, which play a crucial role in regulating vasomotor tone, inhibiting platelet aggregation, and modulating the recruitment and activity of inflammatory cells (Pautz et al., 2021; Tang et al., 2014). Moreover, a decrease in TXB2 levels and an increase in PGI2 serum levels have been found to be associated with the inhibition of platelet aggregation in healthy volunteers (Mitchell et al., 2021).

**FIGURE 7:**
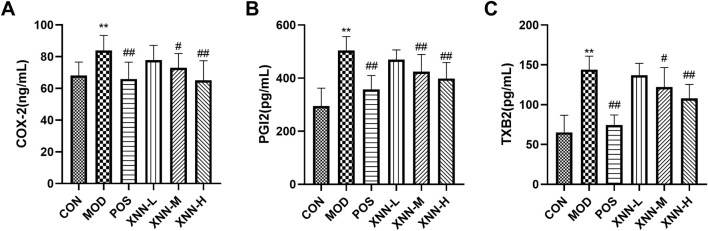
Serum concentrations of COX-2 **(A)**, PGI2 **(B)** and TXB2 **(C)** in rats. cyclooxygenase-2 (COX-2), thromboxaneB2 (TXB2), prostacyclinI2 (PGI2). CON, control group (n = 7); MOD, model of acute microcirculation dysfunction group (n = 12); POS, positive control group (n = 11); XNN-L, *Xinnaoning* capsule low-dose administration group (n = 9); XNN-M, *Xinnaoning* medium-dose administration group (n = 10); XNN-H, *Xinnaoning* high-dose administration group (n = 10). Statistical significance indicates as asterisk (*) when comparing CON group with MOD group, and as hashtag (#) when POS, XNN-L/M/H group with MOD group. **: *p* < 0.01; #: *p* < 0.05; ##: *p* < 0.01.

To validate the aforementioned inference, an Enzyme-Linked Immunosorbent Assay (ELISA) was employed to quantify the levels of COX-2, TXB2, and PGI2 in serum samples. As illustrated in Figure 7, XNN exhibited substantial effects in normalizing the elevated cytokine levels in the serum of AMD rats to baseline values. The results of this verification indicate a significant effect of XNN on the modulation of vasomotor tone, inhibition of platelet aggregation, and regulation of inflammation within this animal model.

The pathogenesis of AMD is complex and involves multiple mechanisms, indicating that a singular pathway-focused intervention may not be sufficient for effective treatment (De Backer et al., 2014). This study is evident that XNN exerts a therapeutic influence on microcirculation disorders by regulating blood viscosity, blood flow, coagulation function, and vascular endothelial function, with the highest dosage demonstrating the most pronounced impact.

## 5 Conclusion

Multiple indicators support the finding that XNN improves microcirculatory system perfusion deficiencies, lowers blood viscosity, delays coagulation in AMD rats, and enhances vasomotor function and vascular permeability. Overall, XNN effectively regulates vascular function in the treatment of microcirculatory dysfunction.

## Data Availability

The original contributions presented in the study are included in the article/Supplementary Material, further inquiries can be directed to the corresponding authors.

## Abbreviations

XNN, *Xinnaoning* capsule; AMD, acute microcirculatory dysfunction; ASA, acetylsalicylic acid; EHI, epinephrine hydrochloride injection; LSCI, laser speckle contrast imaging; ROI, regions of interest; APTT, activated partial thromboplastin time; PT, prothrombin time; TT, thrombin time; FIB, fibrinogen; WBV, whole blood viscosity; PV, plasma viscosity; VEGF, vascular endothelial growth factor; ET-1, endothelin-1; NOS, nitric oxide synthase; ICAM-1, intercellular adhesion molecule-1; VECD, vascular endothelial-cadherin; QC, quality control; UPLC, ultra-high performance liquid chromatography; PCA, principal component analysis; PLS-DA, partial least-squares discriminant analysis; VIP, variable important in projection; SD, standard deviation; FC, fold change; LPC, Lysophosphatidylcholine; COX-2, cyclooxygenase-2; TXB2, thromboxaneB2; PGI2, prostacyclin I2.
